# Economic evaluation of fulvestrant as an extra step in the treatment sequence for ER-positive advanced breast cancer

**DOI:** 10.1038/sj.bjc.6604790

**Published:** 2008-11-18

**Authors:** D A Cameron, D R Camidge, J Oyee, M Hirsch

**Affiliations:** 1 Department of Oncology, Western General Hospital, Edinburgh, UK; 2 Department of Developmental Therapeutics and Thoracic Malignancies Programs, University of Colorado Cancer Center, Aurora, CO, USA; 3 Department of Market Access, Mapi Values, Bollington, UK; 4 Department of Global Health Economics and Outcomes Research, AstraZeneca Pharmaceutical Ltd, Macclesfield, Cheshire, UK

**Keywords:** cost effectiveness, advanced breast cancer, Faslodex, fulvestrant, hormone receptor positive

## Abstract

Drug therapies for advanced breast cancer in hormone-receptor-positive disease include both hormonal and chemotherapies. Current UK practice is to minimise toxicity by using sequential hormonal agents for as long as clinically appropriate. A Markov model was developed to investigate the cost effectiveness of different sequences of therapies, particularly exploring the effects of adding an additional hormonal agent, fulvestrant, to the treatment pathway. A systematic review was undertaken and a panel of seven UK oncologists validated assumptions used for treatment efficacy, treatment pathways and resources used. Fulvestrant was found to be a cost-effective treatment option when added to the treatment sequence as a second- or third-line hormonal therapy for advanced disease. For a cohort of 1000 patients, fulvestrant as a second-line hormone therapy provided an additional 47 life years and 41 quality-adjusted life years (QALYs), at an additional cost of £301 359. This equated to £6500 per life years gained and £7500 per QALY. When used as a third-line option, the fulvestrant arm was dominant providing an increase in health benefit of 27 QALYs for the whole cohort, at a mean overall cost reduction of £430 per patient. Sensitivity analyses showed these results to be robust, demonstrating that fulvestrant is an economically viable additional endocrine option in the United Kingdom for the treatment of hormone responsive advanced breast cancer.

Breast cancer is the most common form of malignancy among women in western Europe, with one in every eight women developing the disease at some point in their lives (American Cancer society, 2006). Despite recent improvements in survival through increased awareness, screening and the wider use of hormonal therapy and adjuvant chemotherapy, advanced breast cancer remains a common clinical problem in the United Kingdom, accounting for 17% of cancer-related deaths in women (Cancer Research UK, 2005).

Hormonal therapies applicable for women whose disease is hormone-receptor positive (HR+) have been introduced. Among these have been the third-generation aromatase inhibitors (AIs), anastrazole, letrozole and exemestane, which are increasingly being used in the adjuvant setting. However, many postmenopausal women relapse after adjuvant hormonal therapy, with a median survival of 17–22 months post-relapse (Scottish Intercollegiate Guidelines Network (SIGN), 2005), demonstrating a need for more effective therapies in this patient population (Robertson *et al*, 2003).

Fulvestrant (Faslodex®) is an oestrogen receptor antagonist with a novel mode of action, which is administered by monthly intramuscular injection. Fulvestrant is licensed in the United Kingdom for the treatment of postmenopausal women with oestrogen-receptor positive, metastatic breast cancer whose disease has progressed or relapsed on or after previous anti-oestrogen therapy. Several clinical studies have confirmed that fulvestrant is at least as effective as AIs in the treatment of advanced breast cancer in patients with HR+ disease (Howell *et al*, 2002, 2004, 2005; Osborne *et al*, 2002; Robertson *et al*, 2003; Ingle *et al*, 2006). In addition, clinical evidence shows that the addition of fulvestrant into a sequence of well-tolerated hormonal therapies can be undertaken without a detrimental effect on responses to subsequent treatments (Vergote *et al*, 2003; Howell *et al*, 2005).

In the absence of international guidelines, common current practice for the treatment of HR+ advanced breast cancer in many countries, including the United Kingdom, consists of sequential administration of hormonal therapies, prolonging the use of endocrine therapy while the disease status allows before switching to more toxic chemotherapies. This requires the availability of a range of therapies with different modes of action, so that tumours developing resistance to one agent are not cross-resistant to another (Steger *et al*, 2005). For postmenopausal women with HR+ breast cancer previously treated with adjuvant tamoxifen, this is often achieved by first using a non-steroidal AI followed by a sequence consisting of a second AI, usually a steroidal drug. However, given the practical difficulties associated with conducting a study of this sort, there are currently no clinical studies investigating the optimal sequencing of hormonal treatments or of using fulvestrant as an additional step within a sequence of hormonal treatments (Tobias, 2004; Laffaioli *et al*, 2005; Bertelli and Paridaens, 2006).

Cost-effectiveness evidence is increasingly required by health-care payers in the United Kingdom to support decisions regarding the inclusion of new drugs on hospital formularies, as well as by national bodies such as the National Institute for Health and Clinical Excellence (NICE) and the Scottish Medicines Consortium. This has provided the rationale to develop a robust health economic model that brings together the best available evidence for the efficacy and safety of different treatment options to estimate relevant overall health outcomes and costs. This model could then be used to investigate whether an additional endocrine treatment step, such as fulvestrant, in a sequence of hormonal treatments for advanced breast cancer is a cost-effective use of the limited UK National Health Service (NHS) resources.

## Materials and methods

### Markov model structure

**Figure 1:**
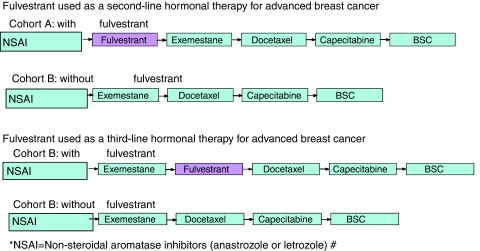
Treatment sequences for Cohort A (with fulvestrant) *vs* Cohort B (without fulvestrant).

In the absence of prospective clinical and health economic data based on a single clinical trial, a Markov cohort simulation model was developed using Microsoft Excel to project longer term clinical and economic consequences for the treatment of postmenopausal women with HR+ advanced breast cancer. The model consists of health states representing each separate line of therapy and death. The model was set up to compare two cohorts of identical patients receiving different treatment sequences of up to five treatments including best supportive care (BSC). Best supportive care was defined as palliative therapy while not on any active treatment and is the package of care given when no active treatments are deemed appropriate for a patient due to treatment toxicity, patient frailty or expected lack of benefit given poor prognosis. Cohort A included the option of fulvestrant as either a second or third therapy in the sequence, whereas Cohort B did not include fulvestrant (Figure 1). Seven UK clinicians (oncologists) were interviewed to obtain information on the most common treatment pathway and clinical and economic parameters which were not available in the literature.

The Markov cycle length defines the unit of time used for assessing the probability of transition between health states within Markov models, and should be based on the natural history of the disease (Briggs and Sculpher, 1998; Philips *et al*, 2006). In our analysis, a Markov cycle length of 28 days was used. The clinicians interviewed felt that a monthly cycle was the most robust option given that most patients are seen in clinic at intervals that are multiples of whole months (1, 2 or 3). In addition, the 1-month treatment cycle was deemed the optimal length to reflect both timing of measurement of disease progression and to maintain adequate simplicity in the model structure.

**Figure 2:**
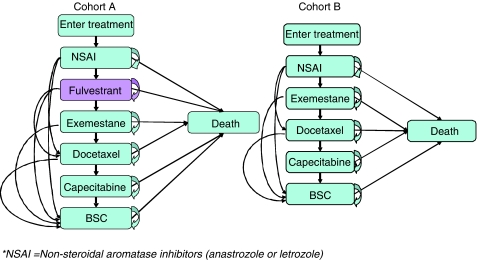
Overview of the sequencing model patient.

At the end of each Markov cycle, patients could either remain on their current line of treatment, experience disease progression and move to another line of treatment or die. The probability of any of these events occurring is dependent on treatment-specific median time to progression (TTP) data and the probability of dying on each treatment. Once patients had experienced a progressive event they could move to any later health states to reflect actual clinical practice. The health states and possible transitions of patients for each cohort are shown in Figure 2.

Each cohort consisted of 1000 postmenopausal women with HR+ advanced breast cancer, assumed to have previously received adjuvant tamoxifen. For computational reasons after 10 years of simulation, all patients in the model were assumed to have died and this was considered to be a reasonable assumption by the clinicians interviewed.

### Clinical data

A systematic review of randomised controlled trials and other experimental studies published between 1998 and 2006 was conducted to identify evidence on TTP for treatments in the selected sequences. Data on the TTP and, where appropriate, the results for a given treatment at a given line of therapy were pooled using meta-analysis and standard statistical methods (DerSimonian and Laird, 1986). A summary of the meta-analysis results for hormonal treatments is presented in Table 1. Time to progression data for chemotherapies were obtained from individual studies that presented sufficient information (Blum *et al*, 1999; O’Shaughnessy *et al*, 2002) (Table 1).

### Proportion dying at each treatment line

The proportions of patients dying at each treatment line were estimated from the clinicians survey, as these data were not available in the literature (Table 2).

### Treatment skipping

The model allowed for the fact that, in clinical practice, not all patients will receive all treatments in sequence. Estimates for treatment skipping were derived from the clinician survey, as these data were not available in the literature:

1. For Cohort B, it was assumed that: 27% would go straight to chemotherapy and 9% straight to BSC from first-line hormonal therapy (AI); 42% would go straight to BSC from second-line hormonal therapy.
2. When fulvestrant is included in the sequence (Cohort A), it was assumed that: 27% would go straight to chemotherapy and 9% would go straight to BSC from first-line hormonal therapy; 27% would go straight to BSC from second-line hormonal therapy; 15% would go straight to BSC from third-line hormonal therapy.

### Costs

The economic evaluation was carried out from the UK NHS perspective and therefore includes only direct medical costs. The direct costs included drug acquisition, health-care resource use associated with treatment administration and monitoring the underlying disease, BSC requirements and treatment of serious adverse events requiring hospitalisation or adverse events with incidence of at least 5%.

The unit drug acquisition costs for hormonal and chemotherapies was derived from British National Formulary (British National Formulary, 2006) (Table 3).

The direct treatment and treatment line-related resource use estimates (e.g., outpatient visits, GP/nurse visits and nurse home visits) were derived from the clinician survey.

The estimated resource use for BSC (health professional consultations, inpatient and hospice stays, radiotherapy and palliative medicine) was also taken from the clinician survey. Costs were calculated by multiplying resource use per patient with the unit cost of resources. All unit costs for resources were taken from nationally published sources, and reported in pounds (£) at 2005 prices (Curtis and Netten, 2005; British National Formulary, 2006).

### Utilities

The specific outcome measures used to evaluate effectiveness in the economic model were life years (LY) and quality-adjusted life years (QALY) gained. The estimation of QALYs gained comparing cohort B with A requires the derivation of a quality of life (utility) weighting on a scale of 0–1 for the different treatment lines, taking into account the impact of toxicities for each treatment in the sequence.

Appropriate utility values were not available from the literature; therefore, the clinicians in the survey were asked to estimate on a 0–100 point visual analogue scale (VAS) the quality of life associated with each line of treatment in the sequence. Their recorded values were then rescaled to 0–1. The resulting mean utilities are presented in Table 4.

### Base case analysis

The cost-effectiveness output was calculated using the incremental cost-effectiveness ratio (ICER), which divides the difference in total health-care costs by the difference in benefits (LYs or QALYs gained) for the two cohorts of patients. The ICERs of below £30 000 per QALY gained are generally considered to be representative of a cost-effective use of UK NHS resources (Rawlins and Culyer, 2004).

In economic evaluations, future costs and benefits are discounted to reflect a personal and societal time preference to delay costs but to have health benefits in the current time period. On the basis of current NICE guidance, an annual discount rate of 3.5% for both costs and benefits (QALY or life years gained (LYG)) was used in the base case economic analysis (National Institute for Clinical Excellence (NICE), 2006).

### Sensitivity and scenario analysis

For the base case treatment sequence comparison, probabilistic sensitivity analysis (PSA) involving 1000 random simulations of the median TTP was performed to quantify the uncertainty around TTP estimates used in the model. The PSA is a technique for testing the robustness of the results from the model by varying multiple parameters simultaneously to see if the cost-effectiveness conclusions are affected. This assessed the level of uncertainty in the cost-effectiveness result and utilised 95% confidence intervals around the TTPs. The results from the PSA were plotted onto a cost-effectiveness plane and a cost-effectiveness acceptability curve (CEAC). The CEAC shows the probability that adding fulvestrant to the treatment sequence is cost effective for a given threshold willingness to pay (WTP) ceiling ratio, that is, the assumed maximum amount a decision maker would be willing to pay for an additional unit of effect (QALY or LYG).

The following one-way sensitivity analyses were performed to further explore the robustness of the model assumptions:

- Discount rate for costs and benefits were varied between 0 and 6%.
- The same efficacy for fulvestrant and exemestane at second and third line was assumed.
- To reflect the multiple potential chemotherapy options in advanced breast cancer, the use of different chemotherapies in the sequence was tested. First, the CMF regimen (cyclophosphamide, methotrexate and fluorouracil) was used instead of capecitabine as a second-line chemotherapy. Second, doxorubicin was used instead of docetaxel as the first-line chemotherapy.
- To reflect uncertainty around chemotherapy effectiveness and use in later lines of therapy an increased median TTP of 6.5 (Chan *et al*, 1999) months and an average number of cycles of 4 was assumed for docetaxel.
- To test the robustness around the number of patients avoiding chemotherapy, a scenario was tested where the number of patients going on to receive docetaxel was the same in both cohorts.

## Results

### Fulvestrant as a second-line hormonal therapy

Cohort A consisted of 1000 postmenopausal women with HR+ advanced breast cancer receiving fulvestrant as a second-line hormonal therapy for advanced disease. The model estimated the total numbers of patients in cohort A who would begin treatment with the following drugs in sequence as: anastrozole/letrozole (1000), fulvestrant (518), exemestane (291), docetaxel (397), capecitabine (258) and BSC (341). For cohort B, without the addition of fulvestrant, the numbers of patients estimated to be receiving treatment at each line in the sequence were as follows: anastrozole (1000), exemestane (518), docetaxel (450), capecitabine (324) and BSC (451). These estimates are based on the probability of progression, estimates used for treatment skipping and proportion of patients expected to die at each treatment line. Hence, in cohort A, 655 chemotherapy regimens were delivered compared with 774 in cohort B. This reduction in the number of patients who received chemotherapy was influenced by the results of the physician survey, which suggested some patients would die while on therapy and others would skip their next therapy. This assumption was varied in a sensitivity analysis.

In the base case analysis, the addition of fulvestrant to the treatment sequence produced an estimated health gain per 1000 women of 56 LYs and 41 QALYs at an additional net cost of £301 359. Per patient this equates to an estimated survival gain of 0.6 months for an additional net cost of just over £300 (Table 5). The resulting ICER was £6500 per LYG and £7300 per QALY. The majority of costs were incurred while on final treatments, while the majority of benefits (QALYs/LYG) were generated through the earlier treatments.

### Fulvestrant as a third-line therapy after two AIs

An alternative scenario positioning fulvestrant as third-line hormonal therapy in cohort A, with exemestane shifting from third- to second-line hormonal therapy, resulted in the sequence with fulvestrant ‘dominating’ the sequence without. The sequence containing fulvestrant both increased health benefits (LYGs and QALYs) and resulted in lower net health-care costs, leading to estimated cost saving of £430 per patient. The difference between this and the base case result occurs because fewer patients actually receive fulvestrant in this scenario. This results in a lower total benefit gain, but also a lower total cost for scenario A, than when fulvestrant is used as second-line therapy option. Ultimately, however, the addition of fulvestrant in both scenarios is cost effective as we see increased levels for benefits of Cohort A over Cohort B, as fewer patients proceed to receiving chemotherapy, which is usually more toxic and expensive than hormonal therapy.

### Sensitivity analyses

The PSA suggested that the addition of fulvestrant into the sequence as either a second- or third-line therapy has a greater than 60 or 70% probability of being viewed as a cost-effective treatment, respectively, given a WTP for a QALY of £30 000. The full cost-effectiveness acceptability curves for the base case analyses have been included as Supplementary Information (Figures 3–4).

Varying the discount rates for costs and benefits between 0 and 6% resulted in a range of incremental cost per QALY gained estimates of £6267–£7813 per QALY gained. The ICER range using LYG as the outcome measure followed a similar pattern.

On the basis of the results of the EFECT study (Gradishar *et al*, 2007), one-way sensitivity analysis assuming the same efficacy (TTP) for fulvestrant and exemestane as a third-line therapy resulted in Cohort A dominating Cohort B, again attaining a greater health gain with a reduction in total costs of £425 per patient. This was very similar to the results for the base case.

For a scenario of CMF as the second chemotherapy choice after second line use of fulvestrant, the ICER was estimated to be £13 673 per QALY gained compared to the cohort B sequence, and ‘dominant’ if fulvestrant was included as third-line hormonal therapy for advanced disease use in the sequence. Where Doxorubicin was used as the first-line chemotherapy choice, the analysis reported an ICER of around £16 000 per QALY where fulvestrant was used as a second-line option, and Cohort A remained ‘dominant’ where fulvestrant was used as the third-line hormonal option.

For sensitivity scenario D, where docetaxel is assumed to have increased efficacy and is given for a reduced number of cycles, the ICERs for second-line use of fulvestrant were £15 773 and £14 423 per LYG and QALY, respectively. Where fulvestrant is added as a third-line therapy, that sequence continues to dominate.

For the final sensitivity analysis where the number of patients receiving chemotherapy on both arms is equalised, this scenario results in cost per QALY's of £24 000 and £19 000, respectively, as fulvestrant is added as a second- and third-line hormonal option. As might be expected, these results are higher than the base case.

## Discussion

Several clinical studies have shown similar efficacy for fulvestrant compared with AIs together with a consistently good tolerability profile (Howell *et al*, 2002, 2004, 2005; Osborne *et al*, 2002; Robertson *et al*, 2003; Ingle *et al*, 2006; Gradishar *et al*, 2007; Chia *et al*, 2008). However, no clinical studies have been conducted that address the issues of effectiveness and cost effectiveness of different treatment sequencing options for women with HR+ advanced breast cancer – a very important question, given standard clinical practice for treating advanced breast cancer. To address this question within a UK context, we constructed a Markov cohort sequencing model to provide the analytical framework to synthesise the available efficacy, safety and cost data for sequences of therapies.

The effects of adding fulvestrant as second- or third-line hormonal therapy for advanced disease were considered. The key results suggested that the option to prescribe an additional hormonal agent was a cost-effective strategy for either of these scenarios when compared with a strategy where prescribing fulvestrant was not an option.

Use of fulvestrant as a third-line hormonal therapy for advanced disease was a highly cost-effective option, as it dominated the comparator option with lower costs and increased benefits. The additional cost effectiveness was mainly derived from the lower total drug acquisition costs when fulvestrant is used as third-line hormonal therapy for advanced disease. This is because fewer patients per cohort would receive the treatment in this setting as compared with second line. However, it should be noted that this model was not designed to examine where to place fulvestrant in the treatment sequence, which should remain a patient-specific clinical question. Rather the analysis suggests that the addition of fulvestrant to the endocrine treatment sequence for appropriate patients is cost effective whether positioned as a second- or third-line endocrine therapy.

In terms of specific benefits, the model estimates that second-line hormonal therapy with fulvestrant will result in fewer patients beginning chemotherapy. This results in economic benefits, with cost savings associated with delayed or reduced use of chemotherapy as well as patient benefits. This is because women with advanced breast cancer spend a greater proportion of time on hormonal treatments, which maintain a higher quality of life than chemotherapies. In addition, there is evidence indicating that some patients have a preference for a monthly injection compared with daily tablets, the formulation of many other hormonal agents (Fallowfield *et al*, 2006). Given an implicit threshold for cost-effective use of NHS resources in the United Kingdom of £20–£30 000, these estimates fall well within the threshold.

The PSA showed that the addition of fulvestrant into the treatment sequence had a relatively high probability of being a cost-effective treatment option despite the multiple data sources used for input parameters. Multiple one-way sensitivity analyses were also conducted on the results to test major assumptions, and all these continued to suggest cost-effective results, confirming the robustness of the model.

Overall, the results suggest that the option of an additional hormonal agent, such as fulvestrant, in the sequence of therapies for advanced breast cancer is a cost-effective treatment strategy. The decision as to when to use fulvestrant in the sequence and in which patients must remain a clinical one; however, it has been shown that when used in appropriate patients, fulvestrant is a cost-effective strategy.

There are a number of possible limitations with the analysis. It may be suggested that a BSC treatment in place of the additional hormonal agent might also be cost effective, as it may also delay and reduce the amount of expensive chemotherapy consumed. At present, there are no data available to fully evaluate this scenario in the model framework given that it is not commensurate with clinical practice. However, it may be postulated that this scenario would not result in the additional QALYs gained that the present results suggest for the fulvestrant arm.

Second, the lack of direct clinical trial data comparing the addition of fulvestrant to a sequence of treatments for advanced breast cancer without fulvestrant may be considered a data limitation. However, the sequencing model is a pragmatic alternative to a complex problem that may represent clinical realities better than would be possible with a single trial. A specific treatment sequence was adopted for the UK context, but the model is fully flexible to compare other treatment sequences. It also provides a framework for synthesising and making best use of a range of data inputs using formal and robust statistical methods, such as meta-analysis, supported by expert clinical opinion, to inform a specific health-care decision problem. The limitations still remain in this particular analyses, for example the chemotherapy options in the model are illustrative rather than comprehensive. However, as sensitivity analysis showed, the specific choice of chemotherapy regimen may not be expected to have a large impact on the cost-effectiveness results. In addition, utility estimates were drawn from clinician opinion on a VAS, rather than from patients. Nevertheless, the estimates are comparable to those reported for advanced breast cancer patients on chemotherapy (Hutton *et al*, 1996).

While several chemotherapy options were examined in the base-case and sensitivity analyses, it should be noted that there are many other options, for example, other taxanes could be used instead of docetaxel. However, the choices in the reported analyses represent some of the extremes of acquisition cost, so assuming a similar level of efficacy for these agents, these data suggest that whichever chemotherapy is used the conclusion of this analysis will hold.

Finally, although this analysis attempts to cover the most common sequences used in the treatment of HR+ advanced breast cancer, the complexity of the treatment and the limitations of the model mean it is inevitable that not all possible sequences will be covered. One such example is a situation where patients initially receive a chemotherapy and are then given hormonal therapy once the disease is ‘controlled’ or the maximum number of cycles is reached. These additional sequences are potentially important and should be considered areas for further research.

However, in general, this model predicts that in clinical practice approximately one-third of all HR+ advanced breast cancer patients may be eligible for fulvestrant in the United Kingdom. For these patients, the analyses suggest that the addition of fulvestrant to the treatment sequence can be considered cost effective and that fulvestrant is an economically viable additional endocrine therapy step for the UK NHS in the treatment of postmenopausal women with hormone-responsive advanced breast cancer.

## Figures and Tables

**Table 1:** Median TTP (pooled) for hormonal and chemotherapy treatment by line of treatment

**Treatment**	**Line of treatment**	**Median TTP (months)**	**95% CI**
Anastrozole (Nabholtz *et al*, 2003; Rose *et al*, 2003)	First	8.50	7.38, 9.62
Exemestane (Lonning *et al*, 2001; Thurlimann *et al*, 2004; Gradishar *et al*, 2007)	First	8.90	7.20, 10.60
Letrozole (Buzdar *et al*, 2001; Tominaga *et al*, 2002; Rose *et al*, 2003)	First	9.40	6.48, 12.32
Exemestane (Lonning *et al*, 2001; Thurlimann *et al*, 2004; Gradishar *et al*, 2007)	Second	4.16	3.47, 4.86
Fulvestrant (Robertson *et al*, 2003; Ingle *et al*, 2006; Gradishar *et al*, 2007)	Second	4.50	2.66, 6.34
Fulvestrant (Gradishar *et al*, 2007)	Third	3.68	3.45, 5.23
Exemestane (Lonning *et al*, 2001; Gradishar *et al*, 2007)	Third	3.72	3.09, 4.35
Docetaxel (O'Shaughnessy *et al*, 2002)	First /second /third	4.2	3.36, 5.04
Capecitabine (Blum *et al*, 1999, 2001)	First /second /third	3.1	2.48, 3.72

**Table 2:** Proportion of patients who progress to death at each treatment line

	**Proportion of patients dying**	
**Treatment line**	**Cohort A (with fulvestrant)**	**Cohort B (without fulvestrant)**
1	0.19	0.19
2	0.23	0.23
3	0.28	0.28
4	0.35	0.35
5	0.5	1.0
6	1.0	—

Source: UK clinicians survey.

**Table 3:** Costs of drug treatments in the sequencing model for the United Kingdom

	**Dose**	**Days each unit prescribed**	**Costs per cycle**
Fulvestrant	250 mg every 4 weeks	28	£348.27
NSAI (anastrazole)^a^	1 mg daily	28	£68.56
Exemestane	25 mg daily	28	£82.88
Docetaxel	100 mg/1.73 m^2^	21	£1,254.00
Capecitabine	Dose 1250 mg/m^2^ twice daily for 14 days, then 7 days interval	21	£296.24

a The cost is similar to letrozole and is assumed that clinical behaviour is most likely to be independent of non-steroidal AI used.

Source: British National Formulary, 2006 (British National Formulary, 2006).

**Table 4:** Utility values for each treatment in the sequence

	**Cohort A (with fulvestrant)**	**Cohort B (without fulvestrant)**
1	0.81	0.81
2	0.73	0.73
3	0.53	0.42
4	0.42	0.42
5	0.35	—
BSC	0.19	0.19
Death	0.00	0.00

Source: UK clinician survey.

**Table 5:** Key results for second and third line use of fulvestrant

	**Second line use of fulvestrant**				**Third line use of fulvestrant**			
	**Cohort A (with fulvestrant)**	**Cohort B (without fulvestrant)**	**Difference**	ICER*	**Cohort A (with fulvestrant)**	**Cohort B (without fulvestrant)**	**Difference**	**ICER**
NSAI (anastrazole/ letrozole)	£1233	£1233	£0		£1260	£1260	£0	
Fulvestrant	£2037		£2037		£755	£755	£0	
Exemestane	£472	£725	−£253		£1296		−£400	
Docetaxel	£4151	£4572	−£421		£4345	£4745	−£400	
Capecitabine	£1120	£1302	−£182		£1198	£1382	−£182	
BSC	£2713	£3593	−£880		£2802	£3857	−£1055	
Cost per patient	£11 725	£11 424	£301		£11 055	£11 424	−£370	
Mean survival (months)	22.3	21.7	0.56		22.34	21.87	0.468	
Life years per patient	1.86	1.81	0.05		1.86	1.82	0.04	
QALYs per patient	1.18	1.14	0.04		1.178	1.142	0.036	
Incremental cost per LYG		—		£6500		—		Cohort A dominant
Incremental cost per QALY gained		—		£7300		—		Cohort A dominant

LYG=life years gained; QALYs=quality-adjusted life years; ICER=incremental cost effectiveness ratio. ^*^ICER values may not be directly derived from the figures presented in the table due to rounding errors. ICER values presented the nearest hundred pounds.
