# Supplementary material for: Economic evaluation of fulvestrant as an extra step in the treatment sequence for ER-positive advanced breast cancer
Source: Br J Cancer. 2008 Nov 18;99(12):1984–90. doi: 10.1038/sj.bjc.6604790 (PMC2607221; doi:10.1038/sj.bjc.6604790)
Supplement: Supplementary Information [file 6604790x1.doc]

Supplementary online material

Figure 3: Cost-effectiveness acceptability curve (CEAC)

Figure 4: Cost-effectiveness acceptability curve (CEAC)
